# Anaphylaxis

**Published:** 1909-10-30

**Authors:** 


### MEDICINE.

### ANAPHYLAXIS.

It has become known that it is sometimes dangerous to repeat the hypodermic injections of an anti-toxic serum at an interval of more than 10 days from the original dose, provided, of course, that a period of several months or longer is not meant by the interval referred to. If a patient has diphtheria and is treated with anti-toxin, and then is found to have a recurrence of the diphtheritic symptoms, say three weeks after the original attack, it might seem good practice to use the anti-toxic serum again. Quite the reverse is the case. Not only is it unwise to use the serum, but it is positively dangerous when 10 days or more have elapsed after the first dose. This danger of repeating the hypodermic injection of an anti-diphtheritic serum is an example of what is known as anaphylaxis. It is not the anti-toxin which is concerned in this pnenomenon, but the proteids of the horse serum, and it is not only horse serum injections that are followed by this undue and even fatal sensitiveness to a repetition after a certain number of days have elapsed, but it seems to be a phenomenon that is common to many proteid substances.

An animal, say a guinea-pig, is inoculated with quite a small dose of complex albuminous substances, such as horse serum, and after an interval of not less than 10 days it is inoculated again with a second dose. We will suppose that the doses in each case are small enough not to produce any symptoms at all if used upon separate guinea-pigs. If the animal that has been inoculated the second time is observed, it will be found that for a time after the second dose it appears none the worse, but then it begins to suffer from nasal irritability, and it vigorously scratches its nose with its fore paws. It becomes exceedingly restless and short of breath, and it also coughs from time to time. Then it becomes paralysed, first of all in its hind legs and then in its fore legs, lies helplessly on its side, and finally develops convulsions which terminate in death. These symptoms may run through their course in so short a time as five minutes, though sometimes they take a good deal longer, and occasionally may even pass off altogether. At a post-mortem examination upon the animal all the organs, except the lungs, which are pale, are found to be congested. Rigidity of the diaphragm is well marked and small hæmorrhages are visible in the abdominal organs and in the wall of the stomach. The death of this animal is the result of anaphylaxis—that is to say, undue sensitiveness to the proteids of horse serum as the result of having had a previous dose of horse serum more than 10 days before.

Anaphylaxis can be produced equally well with milk, with egg albumin, and other proteids of a similar nature. Peptones and albumoses and other similar proteids are less effective.

The refractory period seems to be never less than 10 days, and during this latent period a second inoculation produces none of the symptoms described. While never less than 10 days in its development, it may be longer. Anaphylaxis has been known to last for more than a year. The importance of this in connection with serum treatment is obvious, particularly in cases of chronic infection with bacteria such as streptococci, where it may seem advisable to continue anti-streptococcal serum injections at intervals for weeks or months—in a case of chronic septic endocarditis, for instance.

The explanation of anaphylaxis is a complex problem upon which we do not propose to enter here, but certain main facts about it may be of interest. It has been shown that serum when heated to boiling point before the first inoculation is still able so to affect the animal that anaphylaxis occurs on the second inoculation, provided that the serum used for the second dose has not been heated to boiling point also. If the second dose has been treated as well as the first the symptoms do not appear. It seems, therefore, that that which is present in the first dose to make the animal hypersensitive to the second dose is not destroyed by boiling heat, whilst that which produces the symptoms in the animal as the result of anaphylaxis when the second dose is given is a substance which heat destroys. In other words, serum seems to contain two substances, each of which plays a part in the phenomenon of anaphylaxis. Of these, the first is a thermostabile substance which sensitises the animal or man; the second a thermolabile substance which produces the symptoms.

